# Cardiotoxicity of Anticancer Drugs: Molecular Mechanisms and Strategies for Cardioprotection

**DOI:** 10.3389/fcvm.2022.847012

**Published:** 2022-04-15

**Authors:** Marco Bruno Morelli, Chiara Bongiovanni, Silvia Da Pra, Carmen Miano, Francesca Sacchi, Mattia Lauriola, Gabriele D’Uva

**Affiliations:** ^1^Scientific and Technological Pole, IRCCS MultiMedica, Milan, Italy; ^2^National Laboratory of Molecular Biology and Stem Cell Engineering, National Institute of Biostructures and Biosystems (INBB), Bologna, Italy; ^3^Department of Experimental, Diagnostic and Specialty Medicine (DIMES), University of Bologna, Bologna, Italy

**Keywords:** cardiotoxicity, cardioncology, cardiomyocyte death, cardiomyocyte dysfunction, cardiomyocyte survival, chemotherapy, targeted therapy, cardioprotection

## Abstract

Chemotherapy and targeted therapies have significantly improved the prognosis of oncology patients. However, these antineoplastic treatments may also induce adverse cardiovascular effects, which may lead to acute or delayed onset of cardiac dysfunction. These common cardiovascular complications, commonly referred to as cardiotoxicity, not only may require the modification, suspension, or withdrawal of life-saving antineoplastic therapies, with the risk of reducing their efficacy, but can also strongly impact the quality of life and overall survival, regardless of the oncological prognosis. The onset of cardiotoxicity may depend on the class, dose, route, and duration of administration of anticancer drugs, as well as on individual risk factors. Importantly, the cardiotoxic side effects may be reversible, if cardiac function is restored upon discontinuation of the therapy, or irreversible, characterized by injury and loss of cardiac muscle cells. Subclinical myocardial dysfunction induced by anticancer therapies may also subsequently evolve in symptomatic congestive heart failure. Hence, there is an urgent need for cardioprotective therapies to reduce the clinical and subclinical cardiotoxicity onset and progression and to limit the acute or chronic manifestation of cardiac damages. In this review, we summarize the knowledge regarding the cellular and molecular mechanisms contributing to the onset of cardiotoxicity associated with common classes of chemotherapy and targeted therapy drugs. Furthermore, we describe and discuss current and potential strategies to cope with the cardiotoxic side effects as well as cardioprotective preventive approaches that may be useful to flank anticancer therapies.

## Introduction

The introduction of antineoplastic drugs has been a turning point for prognosis improvement in oncology patients. However, a large number of chemotherapeutic agents have adverse cardiovascular effects, leading to acute or delayed onset of cardiac dysfunction, commonly referred to as cardiotoxicity. Although the definition of cardiotoxicity is not universally accepted, in clinical practice, cardiotoxicity commonly indicates a decline in patients’ cardiac function measured as left ventricle ejection fraction (LVEF). Various organizations and clinical committees defined cardiotoxicity using different threshold changes in LVEF [reviewed in (1)]. Treatment with anthracyclines, namely the chemotherapy class of drugs that generated the most concerns about cardiotoxicity, is associated with an incidence of cardiac dysfunction ranging between 2% and 48% [reviewed in (2–7)]. The Cardiac Review and Evaluation Committee (CREC), a retrospective study aiming at the evaluation of the cardiotoxicity of the anti-HER2 agent trastuzumab with or without concomitant anthracycline treatment, defined cardiotoxicity as a reduction in LVEF of at least 5% to below 55% with concomitant signs or symptoms of congestive heart failure (CHF), or a decrease in LVEF of at least 10% to below 55% without associated signs or symptoms (8). Although the assessment of LVEF is a well-established clinical procedure for the early recognition of cardiotoxic side effects to prevent irreversible cardiac damage and heart failure (HF), a reduction in LVEF may not be an effective parameter to detect a subclinical myocardial dysfunction that subsequently evolves in a symptomatic CHF (9) [reviewed in (1, 10)].

During the last decades, the cardiotoxic effects of several classes of chemotherapy drugs (anthracyclines, fluoropyrimidines, taxanes, and alkylating agents) and targeted therapies (targeting monoclonal antibodies and kinase inhibitors) were documented, and the underlying molecular mechanisms were investigated to suggest and develop potential strategies to avoid or reduce these effects (Table 1). Based on retrospective pathophysiological analysis of cancer patients with HF after chemotherapy, cardiotoxic side effects can be defined as irreversible (type I) or reversible (type II) [reviewed in (11, 12)]. Irreversible cardiotoxicity (type I) is usually observed in anticancer regimes causing injury and loss of cardiac myocytes. These effects are mainly observed after administration of anthracyclines and alkylating drugs, and to a lesser degree with fluoropyrimidines. According to the class of anticancer agents, the underlying mechanisms may involve cardiomyocyte-intrinsic and/or indirect mechanisms. For example, anthracyclines are associated with a high incidence of HF as consequence of irreversible cardiac damages through impairment of cardiomyocyte-intrinsic mechanisms leading to cell death [reviewed in (5, 7, 13–15)]. Despite administration of alkylating drugs and fluoropyrimidines may also cause cardiomyocyte death and thus irreversible cardiac damage, the main mechanism appears to be mediated by a vasculature dysfunction and/or thromboembolic ischemia. However, anticancer agents may also impair cardiomyocyte function without inducing cell death. This type of cardiac dysfunction is typically reversible and is associated with a lower incidence of HF (type II cardiotoxicity). Mechanistically, it has been suggested that reversible cardiotoxicity may be consequent to the deregulation of cardiomyocyte-intrinsic mechanisms and/or alteration of other cardiac populations and extracellular factors, in particular paracrine factors, in turn influencing cardiomyocyte function [reviewed in (4)]. Targeting monoclonal antibodies or tyrosine kinase inhibitors (TKIs) are typically associated with reversible cardiac damages, and their adverse effects derive by the signaling impairment of cardioprotective factors for cardiomyocytes, such as Neuregulin-1 (NRG1), or for other cardiac cell populations, such as vascular endothelial growth factor (VEGF), and platelet-derived growth factor (PDGF) [reviewed in (13, 16)].

**TABLE 1:** Main features and mechanisms of cardiotoxic side effect of chemotherapies and targeted therapies along with mitigating strategies.

Anti-cancer agent	Epidemiology of the cardiotoxicity	Cardiotoxic effect	Cellular and molecular mechanisms of cardiotoxicity	Mitigating strategies
**Anthracyclines** (e.g., doxorubicin, daunorubicin, epirubicin, idarubicin) **and anthracycline analogs** (e.g., mitoxantrone)	Patients without risk factors: <2% of doxorubicin-related heart failure for a cumulative dose of 300 mg/m^2^; 3–5% for a cumulative dose of 400 mg/m^2^; 7–26% for a dose of 550 mg/m^2^; 18–48% for a dose of 700 mg/m^2^ (18, 19). In patients with risk factors cardiomyopathy may occur at low doses of anthracyclines [reviewed in (23, 24)].	Permanent damage due to cardiomyocyte death [reviewed in (34)].	Mitochondrial dysfunction in cardiomyocytes induced by formation of reactive oxygen species (ROS), iron-catalyzed formation of free radicals, lipid peroxidation, and cardiolipin sequestration (44, 45) [reviewed in (6, 14, 34, 38)]. Alteration of mitochondria structural integrity in cardiomyocytes (55). DNA double-strand breaks (DSBs) in cardiomyocytes induced by topoisomerase 2 (Top2) (45, 56) [reviewed in (4, 6)].	**Iron-chelating drug**: dexrazoxane (56, 202, 203, 206) [reviewed in (14, 204, 207)]. **β-Blockers** [reviewed in (16, 37)]: metoprolol (216, 217) carvedilol (220–226) nebivolol (228, 230, 231). **RAAS inhibitors:** ACE-Is such as enalapril, captopril, lisinopril, and ramipril (235–237, 239–241), ARBs such as candesartan and telmisartan (216, 242–245), aldosterone antagonists (251). **Combination of RAAS inhibitors and β-blockers** (33, 247). **SGLT2 selective inhibitors**: empagliflozin (256–259). **INaL inhibitor:** ranolazine (261, 262, 264). **Phosphodiesterase-5 inhibitors**: sildenafil, tadalafil (267–269). **Metabolic agents:** butyric acid (273), β-hydroxybutyrate (276). **Statins** (279–282). **Growth factors:** neuregulins (134, 284, 285, 287), G-CSF (289), erythropoietin (290). **PPARα activators**: fenofibrate (292). **Remote ischemic preconditioning** (293).
		Maladaptive effects on fibroblasts, endothelial cells, vascular smooth muscle cells and immune cells, leading to pathological left ventricular remodeling [reviewed in (67)].	Increased transforming growth factor beta (TGF-β) signaling and myofibroblasts activation [reviewed in (67)]. Increased endothelial cell permeability [reviewed in (67)]. Activation of immune cells [reviewed in (67)].	
**Fluoropyrimidines** (e.g., 5-fluorouracil, capecitabine)	1–19% cardiotoxic events [reviewed in (69, 73, 75)].	Generally reversible coronary artery spasm, although cardiomyocyte death and loss may occur as consequence of coronary artery thrombosis and myocardial infarction [reviewed in (69, 75, 76)], as well as directly through cardiomyocyte-intrinsic mechanisms (77).	Protein kinase C-mediated vasoconstriction in vascular smooth muscle cells (78) [reviewed in (69)]. Reduced oxygen transport capacity of erythrocytes, inducing relative ischemia of the myocardium (79). Increased ROS production in endothelial cells, leading to cell senescence and death, in turn triggering a procoagulant state (77) [reviewed in (69)]. ROS production and induction of cardiomyocyte apoptosis and autophagy (77).	**β-Blockers**, together with **calcium channel blockers, nitrates, and aspirin** [reviewed in (68, 71, 213)].
**Taxanes** (e.g., paclitaxel)	3–20% cardiotoxic events (81, 82) [reviewed in (84)].	Mild, primarily QT interval prolongation, followed by bradycardia and atrial fibrillation (82).	Hypersensitivity reaction with massive histamine release with consequent disturbance of the conduction system and arrhythmia (82). Increased ROS production by cardiomyocyte mitochondria, collapse of mitochondrial membrane potential and opening of mitochondrial permeability transition pore (91).	**Anti-inflammatory**: glucocorticoids [reviewed in (37, 88, 90)]. **Anti-histamine drugs**: histamine receptor blockers [reviewed in (37, 88, 90)].
	Exacerbate anthracycline-induced toxicity (93).	Increment of anthracycline-induced congestive heart failure (92).	Pharmacokinetic interference of doxorubicin elimination by paclitaxel [reviewed in (94)].	
**Alkylating drugs** (e.g., cisplatin, cyclophosphamide, ifosfamide, mitomycin)	7–32% of patients (96) [reviewed in (97)].	Permanent damage due to thromboembolic events and vascular damage, in turn inducing cardiomyocyte degeneration and necrosis (99) [reviewed in (87)].	Increased platelet reactivity by activation of arachidonic pathway [reviewed in (87)]. Oxidative stress and direct endothelial capillary damage with resultant extravasation of proteins, erythrocytes, and toxic metabolites, in turn causing a damage to the myocardium [reviewed in (99)].	**Amino acids:** taurine (102). **NADPH oxidase inhibitors:** apocynin (101).
		Pro-inflammatory effects leading to pathological left ventricular remodeling (101).	Expression of proinflammatory chemokines and cytokines driven by increased NFkB activation (101, 102).	
**ERBB targeting monoclonal antibodies** (e.g., trastuzumab, pertuzumab) **and tyrosine kinase inhibitors** (e.g., lapatinib, tucatinib)	Cardiotoxicity in 2–5% of **trastuzumab**-treated patients, leading to heart failure in 1–4% of the cases (151–153) [reviewed in (155, 156)]. Limited data regarding the sole **pertuzumab** cardiotoxicity [reviewed in (37, 297)]. The risk of heart failure is increased by the addition of pertuzumab to trastuzumab plus chemotherapy regimes (171). 2–5% LVEF reduction in patients treated with **lapatinib**, and in 1% of patients treated with tucatinib [reviewed in (173)]. Combination of lapatinib with trastuzumab does not increase cardiotoxicity (175).	Generally reversible alteration of cardiomyocyte contractile function {trastuzumab [reviewed in (8, 158)] and pertuzumab [reviewed in (174)].	Inhibition of the signaling activated by Neuregulin-1 (NRG1), a paracrine growth factor released by cardiac endothelial cells [reviewed in (110–112)].	**β-Blockers**: bisoprolol (215). **RAAS inhibitors:** ACE-Is such as perindopril (215). **Combination of RAAS inhibitors (ACE-Is) and β-blockers** (248, 249). **INaL inhibitor:** ranolazine (265, 266). **Statins** (283).
	May exacerbate anthracycline-induced toxicity, reaching 28% of heart failure incidence when **trastuzumab** is **combined with anthracyclines** (165).	Exacerbation of anthracycline-induced permanent damage through increased cardiomyocyte death (140).	Increase in anthracycline-induced ROS accumulation and consequent cardiomyocyte death (167).	**β-Blockers** (214): bisoprolol (216), carvedilol (226). **RAAS inhibitors:** ACE-Is such as lisinopril (226). **Statins** (283).
**VEGFR/PDGFR tyrosine kinase inhibitors** (e.g., sunitinib, sorafenib)	Up to 47% of patients receiving sunitinib treatment experienced hypertension, up to 28% showed LV dysfunction, and 8% developed congestive heart failure [reviewed in (15)].	Generally reversible [reviewed in (37, 76, 193)].	Sunitinib- or sorafenib-induced VEGFR inhibition reduces the production of the vasorelaxant nitric oxide (NO) by endothelial cells, in turn resulting in hypertension. In turn, hypertension may lead to capillary rarefaction, which may cause LV dysfunction [reviewed in (15, 194)]. Sunitinib- or sorafenib-induced VEGFR inhibition reduces angiogenesis resulting in LV dysfunction [reviewed in (15, 194)]. Sunitinib- or sorafenib-induced PDGFR inhibition induces the loss of pericytes, leading to coronary microvascular dysfunction and LV dysfunction [reviewed in (15, 194)].	**SGLT2 selective inhibitors**: empagliflozin (260).
**BCL-ABL tyrosine kinase inhibitors** (e.g., imatinib, ponatinib)	Despite initial fears (196), the rate of cardiotoxicity upon imatinib treatment was shown to be extremely low, with less than 1% of the patients developing heart failure [reviewed in (37, 197)]. More than 20% of patients receiving ponatinib treatment experienced adverse cardiovascular event, 5% developed congestive heart failure [reviewed in (181, 197)].	Generally reversible (181).	Ponatinib-induced cardiotoxic effects were suggested to be consequent to thrombotic microangiopathy and consequent ischemia (199).	**Growth factors:** neuregulins [proof-of-principle study in (200), reviewed in (197)].

Importantly, the comprehension of different cellular and molecular mechanisms by which common classes of chemotherapy and targeted therapy drugs induce cardiotoxic effects is critical for developing efficient strategies for prevention, early detection, and treatment. Several therapeutical approaches have already been proposed to cope with the cardiotoxic side effects of anticancer therapies, including iron-chelating drugs, β-blockers, renin-angiotensin-aldosterone system inhibitors, sodium-glucose cotransporter-2 (SGLT2) inhibitors, late inward sodium current (INaL) selective inhibitors, phosphodiesterase-5 inhibitors, metabolic agents, statins, and growth factors. As future therapeutic goal, moving toward a protective chemoprevention approach, we need well-tolerated drugs that may flank chemotherapy to reduce clinical and subclinical cardiotoxic side effects, without interfering with the action of the antineoplastic treatments (17).

## Cardiotoxicity Mechanisms Associated With Common Classes of Chemotherapy Drugs and Targeted Therapy

### Chemotherapy Drugs

#### Anthracyclines

The anthracyclines, such as doxorubicin, daunorubicin, and epirubicin, are a class of broad-spectrum anticancer drugs extracted from *Streptomyces* bacterium. These compounds are used to treat different adult and pediatric hematologic cancers, such as leukemia and lymphomas, as well as many solid tumors, including breast, stomach, uterine, ovarian, bladder and lung cancers. However, anthracyclines are associated with a dose-dependent risk of cardiomyopathy and HF [reviewed in (2–7)]. Specifically, in the absence of risk factors, **doxorubicin** is tolerated up to a cumulative dose of 300 mg/m^2^, with a rate of HF of less than 2% (18). Retrospective studies have shown that an estimated 3–5% of patients, without other risk factors, would experience doxorubicin-related HF at a cumulative dose of 400 mg/m^2^, increasing at 7–26% and 18–48% for a dose of 550 and 700 mg/m^2^, respectively (18, 19). Based on these evident cardiotoxic effects, high-dose treatments with anthracycline are no longer administrated, but sub-acute and chronic cardiac effects are still a clinical problem. The use of second-generation analogs of doxorubicin, namely **epirubicin or idarubicin**, exhibits improvements in their therapeutic index, but the risks of inducing cardiomyopathy are not abated [reviewed in (6)]. Mitoxantrone, which is an anthracenedione, an anthracycline analog, can also damage the cardiac muscle cells, thus resulting in cardiac dysfunction (20) [reviewed in (21, 22)].

Importantly, a large body of evidence indicates that cardiomyopathy develops at lower doses of anthracyclines in the presence of risk factors, including hypertension, arrhythmias, coronary disease, combination with other anticancer agents as well as genetic predisposition to cardiotoxicity [reviewed in (17, 23, 24)]. In this regard, among the genetic factors increasing the susceptibility to anthracycline-induced cardiotoxic effects, the role of specific single-nucleotide polymorphisms (SNPs) is emerging [reviewed in (24, 25)]. Indeed, heritability analysis on multiple cell lines unveiled SNPs from 30 genes giving a greater predisposition to daunorubicin-induced cardiotoxicity (26). Specifically, several SNPs associated with anthracycline cardiotoxicity affect genes involved in anthracycline metabolism, transport, or downstream cytotoxic effects. For example, studies on pediatric cohorts enlightened polymorphisms in CBR1 and CBR3 genes (encoding for carbonyl reductases) associated with enhanced cardiotoxicity susceptibility in children with cancer (27), polymorphisms in ABCC1 and ABCC5 genes (encoding for ATP-binding cassette transporters) associated with increased anthracycline-induced cardiac dysfunction in acute lymphoblastic leukemia patients (28, 29), polymorphisms in SLC22A gene (encoding for a solute carrier) (30), as well as polymorphisms in genes playing a role in iron homeostasis (31), and others [reviewed in (25, 32)]. Oppositely, SNPs in endothelial nitric oxide synthase (NOS3) gene have been reported to be cardioprotective in patients upon a high dose of doxorubicin (29).

**FIGURE 1:**
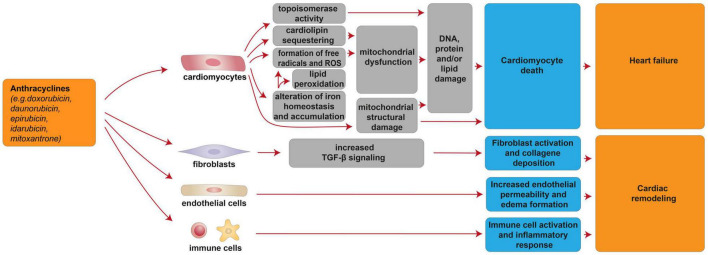
Cellular and molecular mechanisms of the cardiotoxic effects exerted by anthracyclines. Schematic diagram showing the impact of anthracyclines on a multitude of cardiomyocyte-intrinsic mechanisms leading to mitochondrial dysfunction and structural damage and/or DNA damage by topoisomerase activity, in turn leading to cardiomyocyte death and heart failure. Additional mechanisms of anthracycline-induced cardiotoxicity include deregulation of fibroblasts, endothelial, and immune cells, in turn concurring to cardiac remodeling.

Cardiac injury after anthracycline administration occurs with every dose, as documented by the analysis of cardiac-biopsy specimens a few hours after a single dose of anthracycline [reviewed in (7)]. Although the vast majority (98%) of cases of anthracycline cardiotoxicity being detected within the first year after completing the treatment (33), anthracycline-induced cardiotoxicity can also manifest months to years after completing chemotherapy (33). From a pathophysiological point of view, anthracyclines were suggested to induce cardiotoxicity through cardiomyocyte-intrinsic mechanisms as well as other mechanisms involving other cardiac cell types (Figure 1). Importantly, anthracycline-induced cardiac damage may be permanent due to cardiomyocyte death through several biological processes, including apoptosis, autophagy, necrosis, necroptosis, pyroptosis, and ferroptosis [reviewed in (34)]. In this regard, the alteration of mitochondrial function and integrity emerged as a distinctive feature of anthracycline-induced cardiomyopathy [reviewed in (7, 34–39)]. Mitochondria network is well developed in the cardiac muscle, occupying 36–40% of the cardiomyocyte volume and producing around 90% of the cellular energy [reviewed in (40–43)]. Among the complex underlying molecular mechanisms involved in anthracycline-induced mitochondrial dysfunction is worth to mention the formation of reactive oxygen species (ROS), iron-catalyzed formation of free radicals, lipid peroxidation, and cardiolipin sequestering (44, 45) [reviewed in (6, 14, 34, 38)]. In this regard, in cardiac mitochondria, anthracyclines can be reduced by NAD(P)H-oxidoreductases and converted to unstable metabolites, such as doxorubicin-semiquinone radicals, which can react with molecular oxygen (O_2_), producing superoxide anion-free radicals and hydrogen peroxide (${\text{O}}_{2}^{-}$ and H_2_O_2_) (46) [reviewed in (37)]. ROS generated by anthracyclines affect the activity of many mitochondrial enzyme complexes, such as NOSs, NAD(P)H oxidases, catalase, and glutathione peroxidase (GPx), leading to DNA, protein, and lipid damage, and consequently to cardiomyocyte death [reviewed in (39, 47, 48)]. Moreover, anthracyclines, such as doxorubicin, have been reported to impair cardiac iron homeostasis, resulting in its overload in the cardiac tissue [reviewed in (14, 49, 50)]. Accordingly, patients with anthracycline-induced cardiac dysfunction exhibit higher iron levels in cardiac mitochondria, compared to healthy individuals or patients suffering from anthracycline-independent cardiac dysfunction (44). Doxorubicin can, in fact, chelate the free intracellular iron and form iron-doxorubicin complexes, which, in turn, are able to react with O_2_, further increasing the generation of ROS [reviewed in (4, 14, 49, 50)]. In addition, anthracyclines can directly interfere with the main iron-transporting/-binding proteins. For example, doxorubicin can impair cellular iron mobilization, resulting in its accumulation within ferritin (51), and can reduce the expression of the mitochondrial iron exporter ABCB8 (44). Recent studies have also focused on the detrimental role of mitochondrial iron-doxorubicin complexes triggering cardiomyocyte ferroptosis, a kind of programmed cell death dependent on iron and induced by lethal lipid peroxidation (52) [reviewed in (50)]. In this regard, doxorubicin-induced cardiotoxicity in mouse models was shown to be consequent to a decrease in the expression levels of glutathione peroxidase 4 (GPx4), which is a scavenger for lipid peroxides, in turn inducing peroxidation of unsaturated fatty acids and lipids (52). Anthracyclines are also linked to mitochondria damage because of their high affinity to cardiolipin, a mitochondrial membrane phospholipid that is involved in apoptotic pathways [reviewed in (35, 53)]. Mechanistically, doxorubicin sequesters cardiolipin avoiding its anchorage to cytochrome C or lipid-protein interfaces, thus contributing to mitochondrial dysfunction and ROS formation (54) [reviewed in (35, 53)]. Along with the impaired cardiac mitochondrial function, anthracyclines have been demonstrated to alter the structural integrity of mitochondria. Indeed, it has been reported that doxorubicin stimulates the receptor-interacting protein 3 (RIPK3)-induced activation of Ca^2+^-calmodulin-dependent protein kinase (CaMKII), thus triggering the opening of mitochondrial permeability transition pore (MTPT), and ultimately inducing necroptotic cardiomyocyte death (55).

Several lines of evidence have suggested that nuclear damage induced by topoisomerase 2 (Top2) is another pivotal event in anthracyclines’ cardiotoxic effects (45, 56) [reviewed in (4, 6)]. Specifically, doxorubicin intercalates into DNA and interacts with both Top2-alpha (Top2α) and Top2-beta (Top2β), which are enzymes responsible for managing DNA tangles and super-coils. Top2α is highly expressed in proliferating cancerous cells but not in quiescent tissues; therefore, it is considered one of the key molecular targets of anthracycline anti-tumoral effect (56). Cardiomyocyte toxicity stems from the fact that doxorubicin interacts with cardiac Top2-β, the only isoform expressed by adult mammalian cardiomyocytes. Consequently, the Top2β-doxorubicin-DNA complex induces DNA double-strand breaks (DSBs), ultimately promoting cardiomyocyte death (45, 56).

Tumor protein P53 (p53) has also been implicated in anthracyclines’ cardiotoxic response, although its involvement is currently controversial. Indeed, it has been reported that DNA breaks, induced by acute doxorubicin administration, lead to activation of the DNA damage response (DDR) network, in turn activating p53, which ultimately promotes the apoptotic cascade (57) [reviewed in (58)]. Moreover, in response to cell stress, p53 was shown to accumulate in the cytosol and to localize in mitochondria, triggering a series of death-events related to mitochondrial dysfunction, such as the permeabilization of the mitochondrial outer membrane (MOMP), the release of cytochrome C, the opening of the mitochondrial permeability transition pore (PTP), the impairment of mitochondria, and the production of ROS (59–63). Mice depleted for p53 exhibit a less impaired mitochondrial integrity and reduced cardiac dysfunction following doxorubicin treatment [reviewed in (5)]. In addition, doxorubicin-activated p53 has been shown to contribute to metabolic derangement by inhibiting mitophagy events (45) [reviewed in (4)]. As a result of cytosolic accumulation, p53 binds Parkin and abrogates its translocation to damaged mitochondria and their subsequent clearance by mitophagy (64) [reviewed in (5)]. These results support p53 as a key player in anthracycline-related cardiomyopathies (61, 62) [reviewed in (4)]. Nevertheless, other studies unveiled opposite effects depending on the dosage and timing of doxorubicin-induced cellular stress. Indeed, upon low doses of doxorubicin, which more closely recapitulate the clinical settings, it has been reported a protective role of p53, counteracting the late-onset cardiomyopathy and without activation of p53-dependent cell death cascades (65, 66).

In addition to cardiomyocyte-intrinsic mechanisms, anthracyclines exhibit a wide range of maladaptive effects on other cardiac populations, including fibroblasts, endothelial cells, vascular smooth muscle cells, and immune cells [reviewed in (67)]. In particular, doxorubicin administration was shown to increase endothelial cellular permeability, in turn causing edema formation [reviewed in (67)], to induce ROS-dependent activation of transforming growth factor beta (TGFβ) signaling, in turn triggering myofibroblast activation and collagen deposition [reviewed in (67)], and to induce the activation of the innate immune system and inflammatory response [reviewed in (67)]. Overall, these events were suggested to lead to pathological left ventricular remodeling [reviewed in (67)].

#### Fluoropyrimidines

**FIGURE 2:**
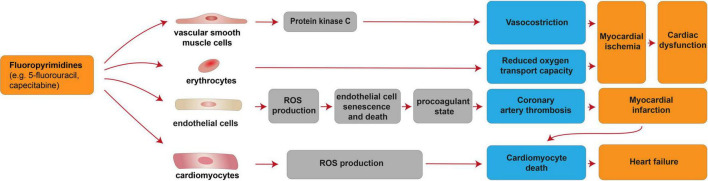
Cellular and molecular mechanisms of the cardiotoxic effects exerted by fluoropyrimidines. Schematic diagram showing the impact of fluoropyrimidines on cardiac dysfunction due to myocardial ischemia induced by deregulation of vascular smooth muscle cells and erythrocytes. Additional mechanisms of taxane-induced cardiotoxicity include heart failure, consequent to cardiomyocyte death induced by cardiomyocyte-intrinsic mechanisms (increased ROS production) or myocardial infarction consequent to coronary artery thrombosis caused by endothelial cell senescence and death.

Fluoropyrimidines exert the second most common cause of chemotherapy-induced cardiotoxicity [reviewed in (68–74)]. This antimetabolite drug class, which includes **5-fluorouracil (5-FU)** and its prodrug **capecitabine**, is incorporated into DNA or RNA, thus acting as cytostatic agent for the clinical treatment of colorectal, breast, gastric, pancreatic, prostate, and bladder cancers [reviewed in (74)]. Fluoropyrimidines are generally well tolerated; nevertheless, 1–18% of the patients receiving fluoropyrimidines experiences cardiovascular toxicity [reviewed in (69–71, 73–75)]. Cardiovascular side effects associated with fluoropyrimidines include a generally reversible coronary artery spasm and myocardial ischemia, although cardiomyocyte death and loss may occur as consequence of coronary artery thrombosis and myocardial infarction [reviewed in (69, 75, 76)], as well as directly through cardiomyocyte-intrinsic mechanisms (77). These adverse effects were suggested to be mediated by vascular smooth muscle cells, erythrocytes, endothelial cells as well as directly by cardiomyocytes (Figure 2). From a molecular point of view, 5-FU was reported to induce protein kinase C-mediated vasoconstriction in vascular smooth muscle cells (78) [reviewed in (69)]. 5-FU was also shown to reduce the oxygen transport capacity of erythrocytes, inducing relative ischemia of the myocardium (79). 5-FU administration was also suggested to induce increased ROS production in endothelial cells, leading to cell senescence and death (77), in turn triggering a procoagulant state and acute thrombotic events [reviewed in (69)]. Finally, direct cardiomyocyte toxicity after fluoropyrimidine administration has also been suggested. Indeed, 5-FU has also been demonstrated to favor ROS production and to induce cardiomyocyte apoptosis and autophagy (77).

#### Taxanes

Taxanes, such as **paclitaxel**, are antimitotic agents that stabilize microtubules in the mitotic spindle, thus blocking cell cycle progression. These chemotherapy drugs are widely employed in cancer treatment, including breast, lung, and ovarian cancers. However, significant toxicities limit the effectiveness of taxane-based treatment regimens (80). Taxane administration is reported to induce cardiotoxic events in 3–20% of the patients (81, 82) [reviewed in (83–85)]. Taxane-induced cardiotoxic effects include QT interval prolongation, followed by bradycardia and atrial fibrillation (82). Because taxane-induced cardiotoxicity appears to be mild in most cases and reversible upon discontinuation of the therapy, no specific agents are recommended for their management [reviewed in (86, 87)].

**FIGURE 3:**
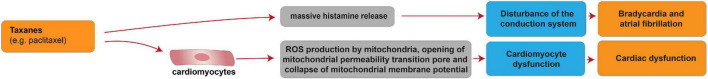
Cellular and molecular mechanisms of the cardiotoxic effects exerted by taxanes. Schematic diagram showing the main cardiotoxic effects of taxanes, namely atrial fibrillation and cardiac dysfunction, as a result of the disturbance of the conduction system or cardiomyocyte dysfunction, respectively.

The underlying cellular and molecular mechanisms of taxane-induced cardiotoxicity are unclear; however, a few hypotheses have been proposed (Figure 3). Among them, hypersensitivity reaction with a massive histamine release and consequent disturbance of the conduction system and arrhythmia has been proposed (82). Hence, the administration of anti-inflammatory (glucocorticoids) and anti-histamine drugs (histamine receptor blockers), is suggested as prophylactic therapy for the management of cardiac anaphylaxis induced by taxanes [reviewed in (37, 88–90)]. Another hypothesis is cardiomyocyte damage through the drug’s actions on subcellular organelles (82). In this regard, taxanes were suggested to increase ROS production by cardiomyocyte mitochondria, the opening of mitochondrial permeability transition pore and the collapse of mitochondrial membrane potential (91).

Among taxanes, paclitaxel has been shown to exacerbate anthracycline-induced toxicity. Indeed, combined treatment with paclitaxel and doxorubicin augmented HF events (92) and increased histopathological alterations of cardiac tissue, with extensive necrosis (93). This effect was suggested to derive from a pharmacokinetic interference of doxorubicin elimination by paclitaxel [reviewed in (94)]. No interaction between doxorubicin and other taxanes (such as docetaxel) has been reported; in line, **docetaxel** showed no increase in cardiac toxicity when combined with doxorubicin [reviewed in (94)].

#### Alkylating Drugs

**FIGURE 4:**
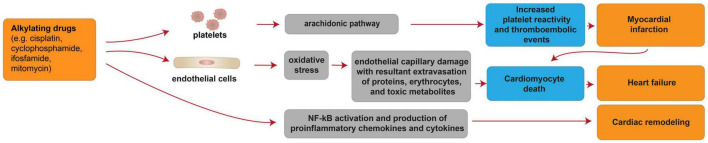
Cellular and molecular mechanisms of the cardiotoxic effects exerted by alkylating drugs. Schematic diagram showing the impact of alkylating agents in promoting heart failure due to cardiomyocyte death consequent to myocardial infarction. Additional mechanisms of alkylating drug-induced cardiotoxicity include heart failure consequent to cardiomyocyte death induced by oxidative stress and cardiac remodeling following activation of pro-inflammatory pathways.

Alkylating drugs, such as cisplatin, cyclophosphamide, ifosfamide, mitomycin, are crosslinking agents inducing ROS production, DNA damage and apoptosis in cancer cells [reviewed in (95)]. **Cisplatin** is mostly used in combination with other chemotherapy drugs to overcome drug-resistance and reduce toxicity [reviewed in (95)]. Cisplatin-based chemotherapy has been reported to cause cardiovascular diseases, particularly myocardial infarction and angina, in a range of 7–32% of patients (96) [reviewed in (97)]. In patients treated with cisplatin, a long-term unfavorable cardiovascular risk profile was observed, with hypercholesterolemia, hypertriglyceridemia, hypertension and insulin-resistance evaluated after more than 10 years from remission (98). The cardiotoxic effects of alkylating agents may be permanent and a few cellular and molecular mechanisms were suggested to contribute to these processes (Figure 4). Indeed, cisplatin administration has been linked with thromboembolic events associated with platelet aggregation and vascular damage (99) [reviewed in (87)], in turn resulting in cardiomyocyte degeneration and necrosis. The increased platelet aggregation was suggested as a direct consequence of cisplatin on the activation of the arachidonic pathway in platelets [reviewed in (87)]. The endothelial capillary damage was suggested to derive from a cisplatin-dependent increase in oxidative stress (99). Indeed, cisplatin has also been shown to induce oxidative stress in myocardial tissue, with decreased activity of glutathione and antioxidant enzymes (100, 101). The consequence of cisplatin-induced endothelial injury was suggested to be the extravasation of proteins, erythrocytes, and toxic metabolites, in turn causing damage to the myocardium (99). Finally, cisplatin has also been suggested to activate NF-κB in the cardiac tissue (101), in turn increasing the expression of proinflammatory chemokines and cytokines (102). This mechanism was proposed to result in cardiac remodeling (101), and extensive degeneration and fragmentation of cardiac muscle fibers (102).

The alkylating agent **cyclophosphamide** at high doses can cause hemorrhagic cell necrosis and may lead to HF; however, with the lower doses currently used, these side effects are infrequent (103).

### Targeted Therapy

#### ERBB Targeted Therapies

Growth factor receptors of the ERBB family (EGFR/ERBB1, ERBB2, ERBB3, and ERBB4) play a key role in the development and progression of a variety of solid cancers [reviewed in (104–106)]. After the binding of soluble ligands, ERBB kinase receptors arrange in homo- or heterodimer complexes, which activate the tyrosine kinase activity and the consequent signaling events leading to the modulation of cell survival, proliferation, migration, and differentiation [reviewed in (104–107)]. ERBB2 (also known as HER2) receptor is a proto-oncogene frequently amplified and overexpressed in many human cancers. Unlike the other ERBB receptors, ERBB2 is unable to bind ligands but heterodimerizes with other ERBB receptors, stabilizing the ligand interaction with the coupled receptors, enhancing and diversifying the ligand-induced receptor signaling (108) [reviewed in (107)]. Several strategies have been developed to target the key role of ERBB2 signaling in tumor development and progression. Successful approaches are represented by treatment with humanized ERBB2-targeting antibodies (e.g., trastuzumab and pertuzumab) and tyrosine kinase multi-HER inhibitors (e.g., lapatinib, tucatinib, afatinib, neratinib, and dacomitinib), which effectively showed ERBB2 inhibition and tumor regression, particularly in the treatment of mammary carcinomas [reviewed in (109)].

**FIGURE 5:**
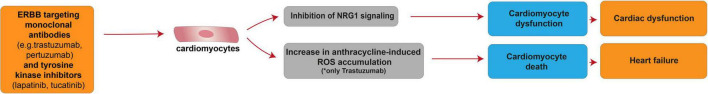
Cellular and molecular mechanisms of the cardiotoxic effect exerted by ERBB targeting monoclonal antibodies and tyrosine kinase inhibitors. Schematic diagram showing the impact of ERBB targeting therapies on cardiomyocyte dysfunction caused by the impairment of Neuregulin-1 signaling. However, in combination with anthracyclines, anti-HER2 monoclonal antibody trastuzumab may also induce heart failure as a consequence of cardiomyocyte death induced by ROS accumulation.

The cardiotoxicity of ERBB2-directed therapeutics is consequent to the inhibition of the signaling activated by Neuregulin-1 (NRG1), a paracrine growth factor released by cardiac endothelial cells featuring pivotal functions in the heart (Figure 5) [reviewed in (110–112)]. NRG1, together with its tyrosine kinase receptors ERBB4, ERBB3, and ERBB2, is essential for heart development (113–115) [reviewed in (110, 116, 117)] and tunes heart regenerative, inflammatory, fibrotic, and metabolic processes (118, 119) [reviewed in (110, 117, 120–122)]. In cardiomyocytes, the most prominently expressed NRG1 receptors are ERBB4 and ERBB2 (123) and NRG1 stimulates fetal/neonatal cardiomyocyte proliferation, hypertrophy, sarcomerogenesis, and survival (114, 115, 124–127) [reviewed in (110, 116, 117, 120, 121)]. ERBB2 forms heterodimers with ERBB4 and is necessary for NRG1-elicited cardiomyocyte proliferation during embryonic and neonatal stages (122, 124). However, cardiac ERBB2 expression levels decline soon after birth in mice, as part of the mechanism leading to cardiomyocyte terminal differentiation, cell cycle withdrawal and loss of cardiac regenerative ability (124) [reviewed in (122)].

Despite low levels described in adulthood, ERBB2 appears to play a role in the prevention of dilated cardiomyopathy. Indeed, mice with ventricular-restricted deletion of ERBB2 exhibited multiple independent parameters of dilated cardiomyopathy, such as chamber dilatation, wall thinning, and decreased contractility (128). Decreased NRG1 signaling in postnatal life is associated with adverse cardiac function and susceptibility to stress [reviewed in (110, 116)]. The expression and activation of ERBB4 and ERBB2 receptors were found lower in myocardium from HF patients (129). In mice subjected to pressure overload, ERBB4 and ERBB2 undergo relevant reduction at mRNA and protein levels with the progression to HF (130).

Conversely, enhanced activity of NRG1 counteracts cardiac remodeling and HF progression [reviewed in (110, 116)]. Systemic administration of NRG1 improves cardiac function following various types of cardiac injuries in adult mice (115, 127, 131, 132) [reviewed in (110, 133)] and HF patients (117, 134–136) [reviewed in (137)].

Cardiac upregulation of ERBB2 was documented upon adverse hemodynamic or other stressful or toxic stimuli, including anthracycline therapies (138, 139). This increase is required to sustain cardiomyocyte survival and cardiac function under stress conditions. Indeed, cardiomyocytes isolated from mice with ventricular-restricted deletion of ERBB2 were more susceptible to anthracycline toxicity, revealing a role for ERBB2 in cardiomyocyte survival upon chemotherapy administration (128). Conversely, cardiac-specific overexpression of ERBB2 in mice has been shown to decrease cardiomyocyte death upon doxorubicin administration (140).

EGFR (also known as ERBB1) is associated with cancer progression and its inhibition *via* monoclonal antibodies (such as cetuximab and panitumumab) or TKIs (such as erlotinib and gefitinib) has been the first strategy evaluated among growth factor receptors targeting therapies (141, 142). Nowadays, EGFR inhibitors are clinically used for the treatment of several solid cancers, including lung, head and neck, colorectal, and pancreatic cancers (142). Although cetuximab-associated cardiotoxicity has been reported in the clinical literature, the incidence of cardiac events in patients remains very low (143, 144).

##### ERBB Targeting Monoclonal Antibodies

**Trastuzumab**, the first ERBB2-targeting humanized monoclonal antibody, binds the extracellular domain IV of ERBB2 receptor leading to the inhibition of ligand-independent heterodimerization between ERBB2 and other ERBB family members (145, 146) [reviewed in (105, 147)]. From a clinical perspective, the cardiotoxicity of monoclonal antibodies targeting ERBB2, such as trastuzumab, is moderate and reversible [reviewed in (148–150)]. Trastuzumab monotherapy is associated with cardiotoxicity in 2–5% of patients, leading to HF in 1–4% of the cases (151–153) [reviewed in (154–157)]. The mechanism of trastuzumab-induced cardiotoxicity appears to be the alteration of cardiomyocyte contractile function without cardiomyocyte death [reviewed in (8, 158)]. Interestingly, Erbb2 gene polymorphisms that alter the ERBB2 protein sequence have been identified, and two of them (Ile 655 Val and Pro 1170 Ala) were associated with an increased risk of cardiotoxicity from trastuzumab therapy (32, 159–164). Importantly, with the concomitant association of trastuzumab and anthracyclines, HF incidence increased to 28% (165, 166). Thus, trastuzumab-mediated blockade of ERBB2 signaling increases anthracycline-induced toxicity. The molecular mechanism underlying this combinatorial phenomenon may be due to the key role of ERBB2 in the management of oxidative stress in the heart: interrupting the neuregulin/ERBB2 axis, which is responsible for the activation of the glutathione reductase system, facilitates the anthracycline-induced accumulation of ROS and subsequent calcium influx, finally leading to caspase activation and cardiomyocyte death (167). Once anti-ERBB2 agents inhibit the ERBB2 protective mechanisms in cardiomyocytes, the doxorubicin oxidative damage was reported to increase (158) [reviewed in (37)].

**Pertuzumab**, a new generation of ERBB2-targeting therapies, is an antibody against domain II specifically designed to inhibit ligand-induced ERBB2 heterodimerization (168, 169). The data regarding the sole pertuzumab cardiotoxicity effects are still limited. Currently, combining trastuzumab/pertuzumab and trastuzumab/lapatinib, in order to induce a dual blockade of HER2, is part of the standard of care (170). In this regard, a recent study reporting a systematic review of eight randomized controlled trials showed that the risk of HF is increased by the addition of pertuzumab to trastuzumab plus chemotherapy therapeutic regimens (171).

##### ERBB Kinase Inhibitors

Tyrosine kinase inhibitors selectively target and inhibit several oncogenic relevant receptor-tyrosine kinases (RTKs), inducing survival benefits in therapies for various hematological and solid cancers [reviewed in (172)]. TKIs include single-targeted and multi-targeted TKIs. A small group of small TKIs, including lapatinib (ERBB2 and EGFR inhibitor), tucatinib (ERBB2 inhibitor), erlotinib (EGFR inhibitor), gefitinib (EGFR inhibitor), afatinib (EGFR, ERBB2, and ERBB4 inhibitor), neratinib (EGFR, ERBB2, and ERBB4 inhibitor), and dacomitinib (EGFR, ERBB2, and ERBB4 inhibitor), has been developed to target ERBB receptors. However, these ERBB blockers can also exert cardiac toxicity in treated patients. In particular, about 2–5% of patients treated with lapatinib displayed a reduced LVEF, and similar effects were reported in 1% of patients treated with tucatinib [reviewed in (173)]. The decline in cardiac function is generally reversible [reviewed in (174)]. Regarding combinatorial anti-ERBB strategies, little is known about the cardiotoxic potential of ERBB2 double blockade with trastuzumab plus lapatinib. Although stronger inhibition of the HER2 pathway using two anti-HER2 drugs was initially expected to result in greater impairment of cardiomyocytes, preclinical tests suggested a possible cardioprotective mechanism exerted by lapatinib. Adjuvant Lapatinib and/or Trastuzumab Treatment Optimisation (ALTTO), a randomized, multi-center, open-label, phase III study of adjuvant lapatinib plus trastuzumab treatment in patients with HER2/ERBB2 positive primary breast cancers (ClinicalTrials.gov, identifier NCT00490139), as well as other clinical trials with double ERBB2 blockade, support the safety of lapatinib plus trastuzumab treatment, since a lower, although not statistically significant, incidence of cardiac events was detected in patients in the trastuzumab plus lapatinib arm. This evidence does not imply that lapatinib has a cardioprotective effect, nor that it should be a preferred option for patients with an increased risk of cardiotoxicity (175).

Afatinib, an ERBB family blocker, approved for the first-line treatment of advanced non-small cell lung cancer (NSCLC) with EGFR mutations, is one of the few TKIs with a low risk of cardiotoxicity [reviewed in (176)]. Finally, cardiac side effects of the irreversible pan-ERBB inhibitor neratinib [reviewed in (177)] were reported neither in phase I clinical studies in solid tumors (178, 179) nor in a phase II trial in advanced HER2-positive breast cancer (179).

#### Multi-Targeted Tyrosine Kinase Inhibitors

**FIGURE 6:**
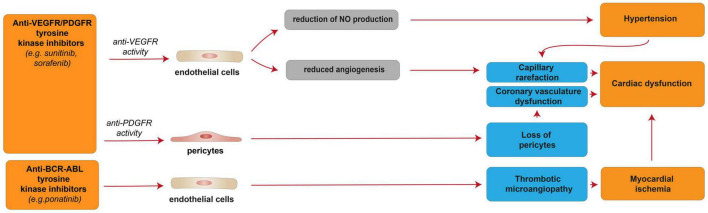
Cellular and molecular mechanisms of the cardiotoxic effects exerted by VEGFR/PDGFR and BCR-ABL and tyrosine kinase inhibitors. Schematic diagram showing the impact of VEGFR/PDGFR and BCR-ABL inhibition, resulting in a reversible cardiac disfunction. Anti-VEFGR activity impairs cardiac function by inducing capillary rarefaction consequent to reduced angiogenesis or hypertension derived from reduced NO production. Anti-PDGFR activity induces cardiac dysfunction by promoting the loss of pericytes, which in turn impairs the coronary vasculature. Anti-BCR-ABL inhibition may results in myocardial ischemia and cardiac dysfunction consequent to thrombotic microangiopathy.

In addition to single- or multi-targeted ERBB family inhibitors (see the previous paragraph), other multi-targeted TKIs were developed to effectively block multiple pathways of intracellular signal transduction. The broad kinase-signaling inhibition of several TKIs, such as sunitinib, sorafenib, imatinib, and nilotinib, includes the vascular endothelial growth factor receptors (VEGFRs), platelet-derived growth factor receptors (PDGFRs), BCR-ABL, and c-KIT. This wide action results in a strong anti-malignancy effect of this class of drugs, although correlated with reversible myocardial dysfunctions with a wide range of severity (180) [reviewed in (37, 181–183)]. Clinical analysis of TKI anti-tumoral therapies shows that compounds with broader off-target effects as kinases inhibitors (lower selectivity in targeting a specific kinase) correlated to higher degree of cardiotoxicity, particularly in case the inhibited kinase plays a role in the maintenance of the cardiovascular system (184–186) [reviewed in (37)]. In this regard, sunitinib, which targets VEGFR/PDGFR and interferes with more than 30 tyrosine kinases; sorafenib, which targets VEGFR/PDGFR and inhibits at least 15 tyrosine kinases, including RAF/MEK/ERK pathway, and ponatinib, which targets BCR-ABL and several other RTKs, are responsible for major clinical concerns related to cardiotoxicity (172) [reviewed in (37, 187, 188)]. Of note, these three compounds (sunitinib, sorafenib, and sonatinib) target VEGF, PDGFR, and c-Kit, namely three tyrosine kinase receptors involved in multiple key functions in the cardiovascular system, whose inhibition is likely the cause of the observed cardiotoxic effects (Figure 6). Particularly, **sunitinib**, which presents an effective multi-targeted inhibition of growth-factor receptors able to reduce the angiogenesis and tumor cell survival/proliferation (182, 189), is considered more cardiotoxic than other anti-angiogenic and TKI drugs (182). Based on clinical studies, 47% of patients receiving sunitinib treatment exhibited hypertension, up to 28% showed LV dysfunction, and 8% developed CHF [reviewed in (15)]. Patients with pre-existing cardiovascular diseases or previous cardio-toxicant exposure show even higher risks [reviewed in (190–192)]. However, cardiac dysfunctions induced by sunitinib and other inhibitors of tyrosine kinases have shown high reversibility; after treatment withdrawal, hypertension and cardiac dysfunction were alleviated or wholly restored (193) [reviewed in (37)]. Indeed, the majority of sunitinib-treated patients were able to carry on with sunitinib therapy following the resolution of cardiovascular events (193). Similarly, reversible cardiotoxicity has been reported upon sorafenib treatment [reviewed in (76)].

The cellular and molecular details of the observed elevated blood pressure and cardiac dysfunction in patients treated with anti-VEGF/PDGFR drugs, such as sunitinib and sorafenib, are not fully understood. Nevertheless, sunitinib- and sorafenib-induced VEGFR inhibition was suggested to reduce the production of the vasodilator nitric oxide (NO) by endothelial cells, in turn resulting in hypertension [reviewed in (15, 194)]. Hypertension is known to lead to capillary rarefaction, which may be responsible for the cardiac dysfunction observed in sunitinib and sorafenib-treated patients [reviewed in (15)]. Indeed, given the high energy dependency, the heart is usually highly vulnerable to any altered blood supply. However, the capillary rarefaction potentially responsible for cardiac dysfunction may also be a direct consequence of reduced angiogenesis following sunitinib- or sorafenib-induced VEGFR inhibition [reviewed in (15, 194)]. Further, sunitinib- or sorafenib-induced PDGFR inhibition was suggested to induce the loss of pericytes, in turn leading to coronary microvascular dysfunction (195) [reviewed in (15, 194)]. Sunitinib, as an off-target effect, has also been suggested to inhibit AMPK activity, in turn inducing energy depletion in cardiomyocytes (184). However, another study found that sunitinib treatment in cardiomyocytes does not affect cellular ATP levels and that myocytes are not protected from sunitinib by pre-treatment with AMPK-activating drug metformin (189).

**Imatinib**, a TKI that inhibits BCR-ABL fusion protein, c-KIT, and PDGFR, is used to treat chronic myeloid leukemia and gastrointestinal stromal cancers. Despite initial fears (196), the rate of cardiotoxicity upon imatinib treatment was shown to be very low, with less than 1% of the patients developing HF [reviewed in (37, 197)]. Nevertheless, the inhibition of CaMKII in adult rat cardiac fibroblasts was shown to reduce the production of mitochondrial superoxide triggered by sunitinib and imatinib treatments (198).

Interestingly, **ponatinib**, a BCR-ABL kinase inhibitor developed to treat patients with imatinib resistance driven by T315I “gatekeeper” mutation, has been associated with a high rate of cardiovascular adverse events. Indeed, more than 20% of patients receiving ponatinib treatment experienced adverse cardiovascular events, and 5% developed CHF [reviewed in (181, 197)]. Of note, these cardiotoxic effects are often reversible with interruption of the therapy (181). The mechanisms of ponatinib-induced cardiotoxic effects are unclear; however, they were suggested to be consequent to thrombotic microangiopathy and consequent ischemia (Figure 6) (199), although cardiomyocyte death was also reported to occur in the zebrafish model (200).

## Strategies to Reduce Anticancer Drug-Associated Cardiovascular Toxicity

Several therapeutical approaches already known in clinical usage have been proposed to reduce cardiotoxicities, such as iron-chelating drugs, β-blockers, renin-angiotensin-aldosterone system (RAAS) inhibitors, SGLT2 inhibitors, late inward sodium current (INaL) selective inhibitors, phosphodiesterase-5 inhibitors, metabolic agents, statins as well as growth factors and hormones [previously reviewed in (201)]. Here we will discuss these classes of drugs, focusing on their mechanisms of action and the therapeutic validity and effectiveness.

### Iron-Chelating Drugs

The iron-chelating drug dexrazoxane has been identified as one of the most promising cardioprotective therapies in these last years and represents the only FDA-approved drug specific for anthracycline-induced cardiotoxicity (202, 203). Dexrazoxane is a pro-drug that rapidly turns into its active form after entering in cardiomyocytes, in turn counteracting the formation of anthracyclines-iron complexes and the subsequent adverse cardiac effects [reviewed in (204)]. Importantly, the development of iron-chelating drugs to prevent anthracycline-induced cardiotoxicity has emerged as an approach of relevant clinical importance in the context of the genetic predisposition of patients suffering from iron-related genetic disorders, such as hereditary hemochromatosis (31, 205). Initially, the cardioprotective functions of this iron chelator were ascribed majorly to its ability to affect iron regulatory proteins and reduce iron accumulation (206) [reviewed in (14, 207)]. However, additional mechanisms have been suggested to drive the cardioprotective activity exerted by dexrazoxane following anthracycline administration. Specifically, dexrazoxane has been shown to modify the topoisomerase 2 (Top2β) configuration preventing its interface with anthracyclines, thereby avoiding the Top2-DNA cleavage complexes (56, 208). Close derivatives of dexrazoxane lacking the interaction with Top2β were found not to be protective in relevant chronic anthracycline cardiotoxicity models (206, 209). Thus, cardioprotective effects of dexrazoxane in chronic anthracycline cardiotoxicity were suggested to derive from the inhibition of the interaction between anthracyclines and Top2β, rather than to its metal-chelating action (209) [reviewed in (210)].

Recently, a study on the cardioprotective effects of dexrazoxane, based on seven randomized trials and two retrospective trials for a total of 2177 patients with breast cancer receiving anthracyclines with or without trastuzumab reported that dexrazoxane reduces the risk of clinical HF and cardiac events in these patients without significantly impacting cancer outcomes (203). Thus, dexrazoxane represents a therapeutical strategy to limit anthracycline cardiotoxicity.

### β-Blockers

β-blockers, also known as beta-adrenergic blocking agents, are a class of drugs that blocks the effects of the hormone epinephrine (adrenaline), causing the heart to beat more slowly and with less force, thus lowering blood pressure. These drugs are predominantly used to manage the reduction in left ventricular ejection fraction (LVEF), preventing symptomatic HF and protecting the heart from a second heart attack event after the first one (secondary prevention) [reviewed in (211, 212)]. The choice of β-blockers as a therapy for cardiac dysfunctions associated with anticancer drugs is mostly based on the dual cardioprotective role exerted by antihypertensive or antiarrhythmic drugs, which preserve cardiovascular function while inhibiting tumor angiogenesis [reviewed in (212)]. β-blockers, together with calcium channel blockers, nitrates, and aspirin are recommended for the management of fluoropyrimidines-induced cardiotoxicity as therapies for angina chest pain, albeit the absence of randomized controlled trials to support their efficacy [reviewed in (68, 71, 213)]. Furthermore, a large number of observations indicate β-adrenergic receptor signaling alterations as a feature of anthracycline-induced cardiomyopathy and in other forms of dilated cardiomyopathies [reviewed in (16, 37)]. A retrospective survey between 2005 and 2010 on 920 breast cancer patients who received anthracyclines and trastuzumab showed an association of continuous β-blocker treatment with a significantly lower incidence of HF events (214). **Bisoprolol**, another second-generation β-blocker, showed stronger efficacy compared to angiotensin-converting-enzyme inhibitor (ACE-I) **perindopril** in attenuating the LVEF decline in patients who received trastuzumab, even though it was unable to avoid left ventricular remodeling (215). However, administration of **metoprolol**, a second-generation β-blocker, did not affect LVEF decline determined by adjuvant, anthracycline-containing regimens with or without trastuzumab and radiation (216) and showed a non-statistically significant reduction in the incidence of anthracycline-induced HF events (217).

In *in vitro* and *ex vivo* set-up, **carvedilol**, a non-selective β- and α1-AR antagonist with strong antioxidant properties, reduced doxorubicin-induced ROS release and cardiomyocyte apoptosis (218) as well as mitochondrial respiration dysfunctions and calcium overloading (219). In rat models of doxorubicin-induced cardiomyopathy, carvedilol showed a significant cardioprotective effect, while **atenolol**, a β-blocker selective for β1-AR and without antioxidant properties, did not, thus suggesting that carvedilol cardioprotective efficacy relies more on its antioxidant properties than on the β-AR blocking action (220). In clinical trials of patients undergoing anthracycline chemotherapy, the prophylactic use of carvedilol decreased the ventricular dysfunction (221–223). In children receiving anthracyclines for acute lymphocytic leukemia, pre-treatment with carvedilol reduced troponin, diastolic dysfunction, and lactate dehydrogenase levels (224, 225). Furthermore, a randomized trial on 468 breast cancer patients treated with anthracyclines with/without trastuzumab showed reduced cardiotoxicity upon carvedilol administration, hence recommending carvedilol as a strategy to reduce trastuzumab interruptions (226).

**Nebivolol** is a cardio-selective β-blocker with mild vasodilating effects due to its interaction with the arginine/NO pathway [reviewed in (227)]. In isolated perfused rat hearts model of anthracycline-induced cardiotoxicity, treatment with nebivolol increased NO levels and significantly reduced oxidative stress, and improved cardiac function (228). Mechanistically, experiments in the rat model suggested that nebivolol administration reduces alterations in cardiomyocyte histomorphometry induced by doxorubicin through modulation of caspase-3, NO synthase (NOS), and TNF-α (229). In randomized placebo-controlled studies, the prophylactic use of nebivolol preserved the cardiac diastolic and systolic function from anthracycline-induced toxicity (230, 231).

To date, the cardioprotective efficacy of β-blockers needs to be further validated in large clinical trials. In addition, in clinical practice, the usage of β-blockers is hampered by their adverse effects in fragile patients, indicating their possible application only in patients with a high cardiotoxicity risk.

### Renin-Angiotensin-Aldosterone System Inhibitors

Several studies showed that alteration of the RAAS has a crucial role in modulating anthracycline-induced cardiotoxicity [reviewed in (232)]. Therefore, the development of RAAS inhibitors, including ACE-Is, angiotensin receptor type 1 blockers (ARBs), as well as aldosterone antagonists, may be effective in the prevention and treatment of anthracycline-induced cardiotoxicity [reviewed in (232, 233)].

**Angiotensin-converting-enzyme inhibitors**, such as enalapril, captopril, lisinopril, and ramipril, impair the conversion of angiotensin I to angiotensin II, with a consequent decrease of angiotensin II receptor type 1 (AT1R) stimulation and its downstream signaling. These compounds have been demonstrated effective in the treatment of hypertension, as well as in reducing mortality in left ventricular dysfunction after myocardial infarction and CHF (234). Preclinical studies in animal models have demonstrated that ACE-Is, such as enalapril, captopril, and lisinopril, can effectively counteract the cardiotoxic effects after single high-dose, multiple low-doses or chronic exposure of anthracyclines (235–238). Mechanistically, ACE-Is’ therapy has been shown to result in the neutralization of ROS damage, reduction of interstitial fibrosis, limitation of intracellular calcium overload, along with improvement of mitochondrial respiration and cardiomyocyte metabolism (235, 236) [reviewed in (232)]. In retrospective clinical analysis, **enalapril** administration to doxorubicin-induced HF children increased cardiac hemodynamic parameters; however, these parameters declined after a few years (239). ACE-I therapy with **ramipril or enalapril** was also shown to induce the recovery of cardiac parameters in patients with doxorubicin-induced cardiac function decline (240). However, no significant improvement in exercise ability or contractile state of pediatric cancer patients receiving anthracyclines was also reported upon enalapril administration, albeit with reduction of left ventricular end-systolic wall stress (241). Clinical trials on HER2-positive breast cancer patients under anthracycline-trastuzumab therapy enlightened cardioprotective effects upon the administration of the ACE-I **lisinopril** (226).

Angiotensin receptor type 1 blockers, such as candesartan and telmisartan, inhibit angiotensin II binding to AT1R. In preclinical rat models, **candesartan** significantly reversed the daunorubicin-induced myocardial pathological changes and cardiac dysfunction (242). Candesartan administration was shown to significantly alleviate the decline in LVEF occurring during adjuvant, anthracycline-containing regimens with or without trastuzumab and radiation (216). Furthermore, in a small prospective study of 49 patients free from cardiovascular diseases and affected by solid cancers, **telmisartan** treatment starting before chemotherapy was able to reduce epirubicin-induced ROS damage by antagonizing the pro-inflammatory signals and reversing the early myocardial impairment (243). Telmisartan administration was also associated with long-lasting (up to 18 months) protection from early and acute myocardial dysfunction in patients treated with epirubicin (244, 245). In contrast, the administration of candesartan was unable to protect against the decrease in left ventricular ejection fraction during or shortly after trastuzumab treatment (246).

Importantly, clinical trials have also shown that a combination of ACE-Is or ARBs and β-blockers has beneficial effects in treating cardiotoxicity induced by anthracyclines and/or anti-HER2 agents. Indeed, the combination of ACE-Is (enalapril) and β-blockers significantly reduced the incidence of cardiac dysfunction along with prevention of the onset of late cardiotoxicity in patients receiving anthracyclines (33, 247). A small phase I trial conducted on 20 women suffering from breast cancer assessed the safety of continuing trastuzumab treatment despite cardiotoxicity onset if patients received ACE-Is and β-blockers following a staggered protocol (248). Another study unveiled the combination of ACE-Is, β-blockers and close cardiac monitoring as an effective strategy for cardioprotection in patients receiving HER2-targeted therapies (249).

Further studies focused on the cardioprotective role of **aldosterone antagonists**, which inhibit the last step of the RAAS and are already known for their beneficial effects on injury-induced cardiac remodeling and fibrosis [reviewed in (250)]. In a small clinical trial, spironolactone has been reported to prevent anthracycline-related cardiac dysfunction in breast cancer patients (251).

### Sodium-Glucose Cotransporter-2 Inhibitors

Sodium-glucose cotransporter-2 selective inhibitors (empagliflozin, canagliflozin, and dapagliflozin) are a group of compounds that have been shown to have protective effects on the progression of HF [reviewed in (252)]. Indeed, EMPA-REG OUTCOME trial demonstrated that empagliflozin reduces major adverse cardiovascular events, cardiovascular death, and hospitalization rates for HF (253). Similarly, EMPEROR-Preserved trials found a reduced risk of HF hospitalization for 9718 patients with HF treated with empagliflozin (254). In a new systematic review meta-analysis of seven studies, for a total of 5,150 HF patients, empagliflozin was effective in reducing cardiovascular death or hospitalization for worsening HF condition (255). Therefore, SGLT2 inhibitors represent a promising treatment for chronic HF patients.

Recently, the potentially protective effects of SGLT2 inhibitors on the cardiac dysfunction induced by chemotherapies and targeted therapies were also investigated in preclinical studies in animal models. In this regard, protective effect by empagliflozin against anthracycline-induced cardiac impairment, diastolic dysfunction, and maladaptive cardiac remodeling has been documented (256–259). Mechanistically, empagliflozin was suggested to reduce ferroptosis, inflammatory response (NF-κB signaling), apoptosis, and fibrosis induced by doxorubicin through the involvement of NLRP3 and MyD88-related pathway (257, 258). A recent pre-clinical study reported that empagliflozin can also improve the cardiac dysfunction induced by anti-VEGFR/PDGFR multi-TKI sunitinib, *via* regulation of cardiomyocyte autophagy, in turn mediated by the AMPK-mTOR signaling pathway (260).

### Late Inward Sodium Current Inhibitors

Selective inhibitors of late inward sodium current (INaL), such as ranolazine, have proven effective in treating experimental HF in several experimental models of cardiac dysfunction given its antiarrhythmic, anti-ischemic, and ATP-sparing features. Experimental evidence suggests that anthracyclines indirectly induce INaL hyperactivation, resulting in cytosolic calcium overload (261–263). INaL hyperactivation contributes to mitochondrial calcium depletion and dysregulation that, in turn, triggers mitochondrial ROS generation (oxidative stress), as well as NAD(P)H and ATP depletion (energetic stress); as a result, these events lead to cardiomyocyte impairment, diastolic dysfunction, and HF progression (261, 262, 264). Importantly, in animal models of doxorubicin-induced cardiotoxicity, ranolazine administration attenuated diastolic cardiac dysfunction and prevented worsening of systolic function by reducing oxidative stress and cardiomyocyte functional derangements (261, 262, 264). Moreover, ranolazine limited trastuzumab-induced cardiac dysfunction in mice by acting as a regulator of cardiac redox balance (265). In a very small randomized clinical study on 24 low-risk patients with diastolic dysfunction induced by anthracycline-based or fluoropyrimidine-/platinum-based therapies, patients were treated for 5 weeks with ranolazine or standard therapy, observing a complete recovery from diastolic dysfunction in all subjects in ranolazine group (12 patients) (266). Thus, the therapeutic use of this drug is promising, although needs validation in large clinical trials specific for each type of chemotherapy.

### Phosphodiesterase-5 Inhibitors

Phosphodiesterase-5 inhibitors, such as sildenafil and tadalafil, were demonstrated to induce cardioprotective effects in animal models affected by doxorubicin cardiac toxicity (267–269). **Sildenafil** demonstrated cardioprotective activity against anthracycline-induced cardiac dysfunction by inducing the opening of mitochondrial K_*ATP*_ channels, leading to preserving mitochondrial potential and functions, myofibrillar integrity, and preventing cardiomyocyte apoptosis (267). The cardiac effects of sildenafil were also suggested to be dependent on the NO-signaling pathway since its protective activity was abolished by both L-NAME (inhibitor of NOS) and 5-hydroxydecanoate (inhibitor of ATP-sensitive K+ channels) (270). **Tadalafil’s** effects on cardiotoxicity reduction, instead, were suggested to be mainly due to NO-mediated increases of protein kinase G (PKG) activity and cGMP signaling, which is significantly reduced by doxorubicin administration (268, 269).

### Metabolic Agents

**Butyric acid**, a short-chain fatty acid produced daily by the gut microbiota, has proven beneficial in models of cardiovascular diseases (271) [reviewed in (272)]. A novel butyric acid derivative, phenylalanine-butyramide (FBA), has been shown to protect animal models from doxorubicin-induced cardiotoxicity by decreasing oxidative stress and improving mitochondrial function (273). Of note, FBA prevented doxorubicin-induced cardiomyocyte apoptosis, left ventricular dilatation, and fibrosis (273).

Another metabolic agent, **β-hydroxybutyrate (BHB)**, produced by fatty-acid oxidation in the liver under the fasting state, was shown to play a cardioprotective role in diabetic and HF with preserved ejection fraction (HFpEF) mouse models, when administrated as a dietary supplement or directly injected (274, 275). Interestingly, BHB was also reported to induce protection against anthracycline-induced cardiac function decline and partially reverted the maladaptive remodeling, characterized by increased cardiomyocyte size and decreased fibrosis (276). *In vitro*, BHB administration reduces oxidative stress and ameliorates mitochondrial functions, decreasing cardiomyocyte cell injury and apoptosis (276).

### Statins

Statins reduce cholesterol synthesis by inhibiting the enzyme HMG CoA reductase. However, statins have emerged as pleiotropic factors playing a positive role on the cardiovascular system, including ROS production and oxidative stress, and the consequent cardiac mitochondrial dysfunction [reviewed in (277, 278)]. Importantly, the treatment of breast cancer patients undergoing anthracycline-based therapy with statins has been reported to be associated with a lower risk for HF and to prevent the decrease of the left ventricular ejection fraction (279–282). A similar cardioprotective activity of statins was reported for trastuzumab-based therapies (283).

### Growth Factors

Administration of the growth factor **Neuregulin-1 (NRG1β)** has been shown to improve cardiac function following injury in adult mice (127, 134) [reviewed in (110, 133)] and in HF patients (135, 136) [reviewed in (137)]. Importantly, administration of NRG1β has also been shown to protect cardiac myocytes from anthracycline-induced apoptosis (134, 167, 284, 285) [reviewed in (286)]. Further, NRG1 administration in the zebrafish model was reported to reduce cardiomyocyte apoptosis induced by the multi-TKI ponatinib (200) [reviewed in (197)]. However, NRG1β is not clinically relevant as a therapy for cardiomyopathy induced by anticancer drugs because of its well-established cancer-promoting role. To solve this issue, an engineered bivalent NRG1 (NN), which preferentially induces ERBB4 homo-dimer formation in cardiomyocytes, has been developed and shown to protect against doxorubicin-induced cardiotoxicity, maintaining the same cardioprotective properties of NRG1 but with reduced pro-neoplastic potential (287). Nevertheless, although up to now there is no evidence in the literature about detrimental side effects in response to bivalent NRG1, NN has not been recruited into a clinical trial yet. Further studies are therefore recommended to assess if this combinatorial treatment is sufficient to mitigate the cardiotoxic side effects of chemotherapeutic agents.

**Granulocyte colony-stimulating factor (G-CSF)** is a hematopoietic growth factor that affects proliferation and differentiation, especially of progenitors of the neutrophil and granulocyte lineages, therefore it is currently used clinically in combination with doxorubicin to counteract doxorubicin-induced myelosuppression (288). Interestingly, a role for G-CSF has also been suggested in doxorubicin-induced cardiomyopathy. Indeed, an attenuation of cardiomyocyte atrophic degeneration and a decrease of myocardial fibrosis have been reported after G-CSF administration in doxorubicin-treated mice (289). Intriguingly, G-CSF was suggested to exert an anti-atrophic and anti-inflammatory activity directly on cardiomyocytes (289).

Among stromal cells, the beneficial role of endothelial progenitor cells (EPCs) has emerged to counteract the cardiotoxicity of cancer therapies. For example, **erythropoietin (EPO)** has been shown to promote angiogenesis by increasing the number of EPCs, thereby improving cardiac function after doxorubicin treatment (290).

### Other Strategies

A few other strategies were suggested to reduce the adverse cardiovascular side effects of common chemotherapies and targeted therapies. In this regard, the sulfur-containing amino acid **taurine** (2-aminoethanesulfonic acid) has been shown to exert beneficial effects in CHF, ischemic heart disease, hypertension, atherosclerosis, and diabetic cardiomyopathy (291). Intriguingly, taurine was also shown to reduce cisplatin-induced cardiotoxicity by suppressing the generation of ROS, ER stress, and inflammation (102). **Apocynin**, a specific NADPH oxidase inhibitor, has been shown to reduce cisplatin-induced oxidative stress, inflammation and apoptosis (101).

Preclinical studies demonstrated that **fenofibrate**, a PPARα activator, counteracted doxorubicin-induced cardiotoxicity in mice by increasing circulating EPCs, stimulating cardiac NO activation and inducing the production of pro-angiogenic factors such as SDF-1 and VEGF (292).

Besides molecular strategies, **remote ischemic preconditioning (RIPC)**, which consists of reversible repetitive interruptions in blood flow, ischemia, and reperfusion, seems a good approach to reduce anthracycline-induced cardiotoxicity (293). Indeed, large animals, subjected to RIPC before each doxorubicin injection, have shown a preserved cardiac contractility and mitochondrial integrity, concomitantly with a higher cardiac performance and reduced fibrosis (293).

## Conclusion and Future Perspectives

Although anticancer therapies greatly improve survival and quality of life of oncological patients, their negative impact on cardiac well-being is a very critical issue. In addition to common risk factors, such as age, hypertension, arrhythmias, and coronary disease, it has emerged the identification of genetic variants related to an increased predisposition to cardiotoxicity of chemotherapies and targeted therapies, in particular for anthracyclines and anti-HER2 therapies. Thus, the development of individualized treatments, based on the forecast of the cardiotoxic side effects, may acquire a considerable clinical relevance for the future perspective. Importantly, the cellular and molecular mechanisms mediating the cardiotoxicity of common classes of chemotherapy and targeted therapy drugs are emerging, providing a rationale for the development of novel strategies for cardioprotection. Recent clinical trials have tested multiple cardioprotective drugs, highlighting the ability of some of them in counteracting or limiting the cardiotoxic effects of anticancer treatments. However, many of these therapeutic strategies still have certain limits and need some precautions. Among them, the lack of validation in large clinical trials, the underlying molecular mechanisms still not fully understood, as well as the risk-benefit controversies. In this regard, it is extremely important to take into account the tolerability of the adverse effects that these therapies may entail, including fatigue and dizziness, in patients already fatigued by antitumoral therapy.

Despite multiple cellular and molecular mechanisms being suggested to mediate the cardiotoxic effect of anti-cancer drugs, cardiomyocyte death has emerged as the major cause of long-term irreversible cardiac disfunction. These important side effects have been documented for anthracyclines, fluoropyrimidines, and alkylating drugs. This is because lost cardiomyocytes cannot be efficiently regenerated due to the very low ability of the adult mammalian heart to produce new cardiomyocytes (294, 295) [reviewed in (296)]. Although the cytotoxic effect of anticancer treatments resides on a wide range of biological mechanisms, the development of strategies aiming at increasing cardiomyocyte survival is thus encouraged to reduce anticancer drug-induced cardiomyocyte death and the consequent permanent damage. In the future, the administration of cardiomyocyte survival factors flanking chemotherapy and targeted therapies should be further explored. In this regard, a plethora of factors and signaling pathways has been shown to trigger endogenous cardiomyocyte proliferation for cardiac regenerative strategies [reviewed in (296)], thus their modulation may be also explored for cancer patients with permanent damage by anticancer drugs. Some of these factors also regulate cardiomyocyte survival, thus their modulation may be tested as a preventive strategy to reduce permanent cardiotoxic effect of anticancer drugs. Obviously, the potential interfering with the action of the antineoplastic treatments should be carefully evaluated.

In conclusion, cardiovascular adverse effects resulting from antineoplastic therapies are important concerns for the health of cancer patients and could question the choice of undertaking or interrupting treatments. Nowadays, some drugs have been clinically tested to counteract the cardiotoxicity related to anticancer care, and we here propose a further evaluation of factors that up to now are mainly known for their role in cardiomyocyte proliferation and survival, as promising strategies for protection and/or regeneration of the cardiac tissue. Moreover, an increasing synergistic effort would be required for the oncologic and cardiologic research fields to assure cancer patients a long-term relapse-free survival and high-quality cardiovascular health.

## Author Contributions

All authors significantly contributed to the writing of the manuscript.

## Conflict of Interest

The authors declare that the research was conducted in the absence of any commercial or financial relationships that could be construed as a potential conflict of interest.

## Publisher’s Note

All claims expressed in this article are solely those of the authors and do not necessarily represent those of their affiliated organizations, or those of the publisher, the editors and the reviewers. Any product that may be evaluated in this article, or claim that may be made by its manufacturer, is not guaranteed or endorsed by the publisher.
